# Merging Lignin and Glycerol Carbonate Valorization Toward the Green Synthesis of *β*‐Adrenergic Blocker Esmolol

**DOI:** 10.1002/cssc.202501540

**Published:** 2025-12-14

**Authors:** Antonio A. Castillo‐Garcia, Katalin Barta

**Affiliations:** ^1^ Institute of Chemistry University of Graz Graz Austria

**Keywords:** amination, glycerol carbonate, hydrogen borrowing, lignin, pharmaceuticals

## Abstract

Producing nitrogen‐containing chemicals through the direct combination of by‐products readily available from agricultural waste, including renewable aromatic building blocks from lignin, is a highly attractive approach for sustainable biorefining processes. Here, we describe a novel synthetic/catalytic route toward the production of the highly valuable *β*‐adrenergic blocker esmolol. Our strategy consists of: 1) Reductive Catalytic Fractionation (RCF) of sugarcane lignocellulose mediated by copper porous metal oxides (Cu_20_PMO) in MeOH, leading to the in situ formation of methyl 3‐(4‐hydroxyphenyl) propionate (**1H**) with good selectivity (>70%), followed by 2) the selective catalytic amination of glycerol carbonate (**GlyC**) with isopropyl amine via the borrowing hydrogen strategy, and 3) the subsequent utilization of the obtained amine intermediate as a phenol alkylating agent in combination with **1H** to afford the desired *β*‐adrenergic blocker esmolol (**1Ha**).

## Graphical abstract

An atom‐economic methodology toward the synthesis of β‐adrenergic blocker esmolol (**1Ha**) from sugarcane bagasse has been developed. The depolymerization of sugarcane via a lignin‐first reductive catalytic fractionation catalyzed by Cu_20_PMO, enables the selective sourcing of methyl(3‐ (3‐hydroxy‐4‐methoxyphenyl)propionate (**1H**). On the other hand, the catalytic amination of glycerol carbonate (**GlyC**) leads to the intermediate **GlyCa**. Finally, the combination of these two bio‐based platform chemicals allows a clean synthetic pathway toward **1Ha** with only H_2_O and CO_2_ as the main side products.

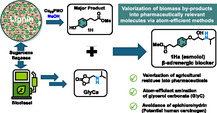

## 1 Introduction

**FIGURE 1:**
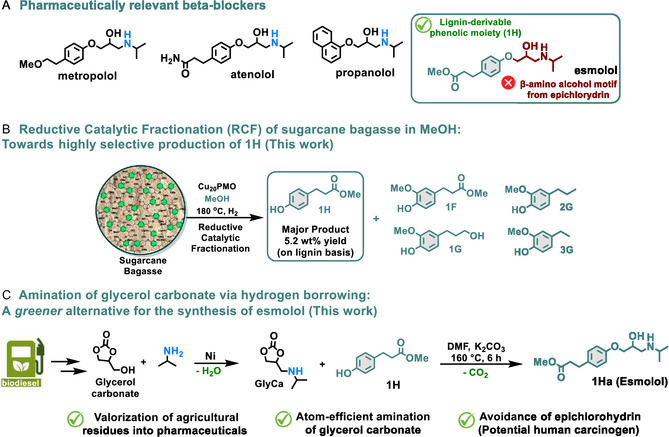
An overview toward the production of esmolol from lignin‐derivable methyl 3‐(3‐hydroxy‐4‐methoxyphenyl) propionate (**1H**) and glycerol carbonate (**GlyC**). (A) Selected examples of relevant beta‐adrenergic blockers. (B) Comparative analysis of RCF procedures for herbaceous feedstocks in MeOH mediated by Ni/C (Ref. [16] and [19]) and Cu_20_PMO (this work). (C) A clean strategy for the synthesis of esmolol via amination of **GlyC** and subsequent reaction with **1H**.

*β*eta‐adrenergic blockers are pharmaceuticals recurrently employed for the treatment of heart‐related diseases, including hypertension, ischemic heart disease (angina pectoris, myocardial infarction), supraventricular arrhythmias, among others [1, 2, 3]. Structurally, conventional *β*‐adrenergic blockers such as propranolol [4], metropolol [5], atenololol [6], and esmolol [7] are substituted phenols displaying *β*‐amino alcohol scaffolds (Figure 1A) [8], thus, the application of biomass‐derived phenolic building blocks represents a truly sustainable alternative for their synthesis [9].

Interestingly, the chemical structure of esmolol [7, 10, 11, 12, 13] (USD $83.88 per gram) [14] contains the phenolic methyl 3‐(4‐hydroxyphenyl) propionate (**1H**), which can be entirely obtained from lignin‐derived *p*‐coumaric acid, predominantly found in numerous herbaceous feedstocks [15] and readily available in agricultural residues [16], namely corn stover [17], wheat straw [18], bamboo [19], miscanthus [20], or sugarcane bagasse [21], in combination with ferulic acid. In the particular case of sugarcane bagasse, the stronger interaction between ferulic acid and hemicellulose facilitates the selective extraction of *p*‐coumaric acid during alkaline treatment [22, 23], enabling further valorization into valuable compounds such as L‐tyrosine [24] or the in situ esterification toward the formation of methyl coumarate [25]. In that respect, the valorization of native sugarcane bagasse into aromatic esters, including **1H**, has been earlier proposed by Doherty and coworkers through a one pot oxidation‐hydrogenation sequence, using atmospheric pressure of O_2_ and MeOH as solvent. However, the low selectivity displayed, together with the usage of high temperatures (250°C) and precious metal‐based Pd/C are nonetheless disadvantageous [26].

Alternatively, the reductive catalytic fractionation (RCF) of herbaceous feedstocks has been efficiently carried out employing earth‐abundant metal catalysts, e.g., Ni/C, yielding monomer fractions containing **1H** as the main component [17, 18, 19, 20]. Intriguingly, the earth‐abundant copper porous metal oxide catalyst (Cu_20_PMO) has displayed a remarkable efficiency for the deconstruction of softwood and hardwood lignocellulose with high selectivity to propanol‐functionalized guaiacols and syringols under mild conditions [27, 28, 29], however, the application for the catalytic deconstruction of herbaceous materials has not been explored (Figure 1B).

The sustainable synthesis of beta‐blocker esmolol (**1Ha**) was developed in this work. We initially chose sugarcane bagasse, which is characterized by a higher lignin content (ca. 30%) compared to other herbaceous feedstocks, to optimize the selective formation of methyl(3‐(3‐hydroxy‐4‐methoxyphenyl)propionate (**1H**) via RCF. Overall, our strategy consists of (a) the catalytic depolymerization of sugarcane bagasse via a lignin‐first RCF approach under mild conditions, mediated by Cu_20_PMO and (b) the efficient incorporation of the *β*‐amino alcohol motif through phenol substitution using bio‐derivable glycerol carbonate (**GlyC**) as green alkylating agent, therefore avoiding the traditional use of toxic and potentially carcinogen epichlorohydrin, contemplating the prior catalytic amination of **GlyC** with isopropyl amine following the atom‐efficient borrowing hydrogen methodology (Figure 1C) [30].

## 2 Results and Discussion

### 2.1 Selective Formation of 1H from Sugarcane Bagasse via Cu‐Catalyzed Reductive Catalytic Fractionation

**FIGURE 2:**
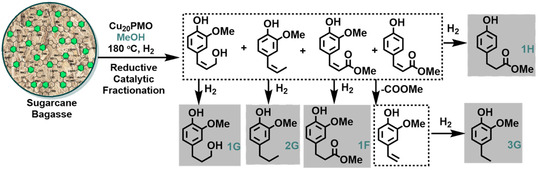
Cu_20_PMO‐mediated reductive catalytic fractionation (RCF) of native sugarcane bagasse in MeOH.

Throughout the last years, our group has developed the Cu_20_PMO‐mediated lignin‐first RCF of hardwood and softwood [27, 28, 29, 31, 32, 33]. In the case of softwood lignocellulose, monomer yields of ≈10−12 wt% are commonly achieved, obtaining 4‐propanol guaiacol (**1G**) with exceptional selectivity (>90%). Hence, at the core of this approach is the use of a non‐noble‐metal catalyst, copper‐doped porous metal oxide targeting methyl 3‐(4‐hydroxyphenyl) propionate (**1H**) as major compound among other lignin‐derived monophenolics, namely methyl 3‐(3‐hydroxy‐4‐methoxyphenyl) propionate (**1F**), 4‐propanol guaiacol (**1G**), 4‐propyl guaiacol (**2G**), or 4‐ethyl guaiacol (**3G**) (Figure 2) [20].

**FIGURE 3:**
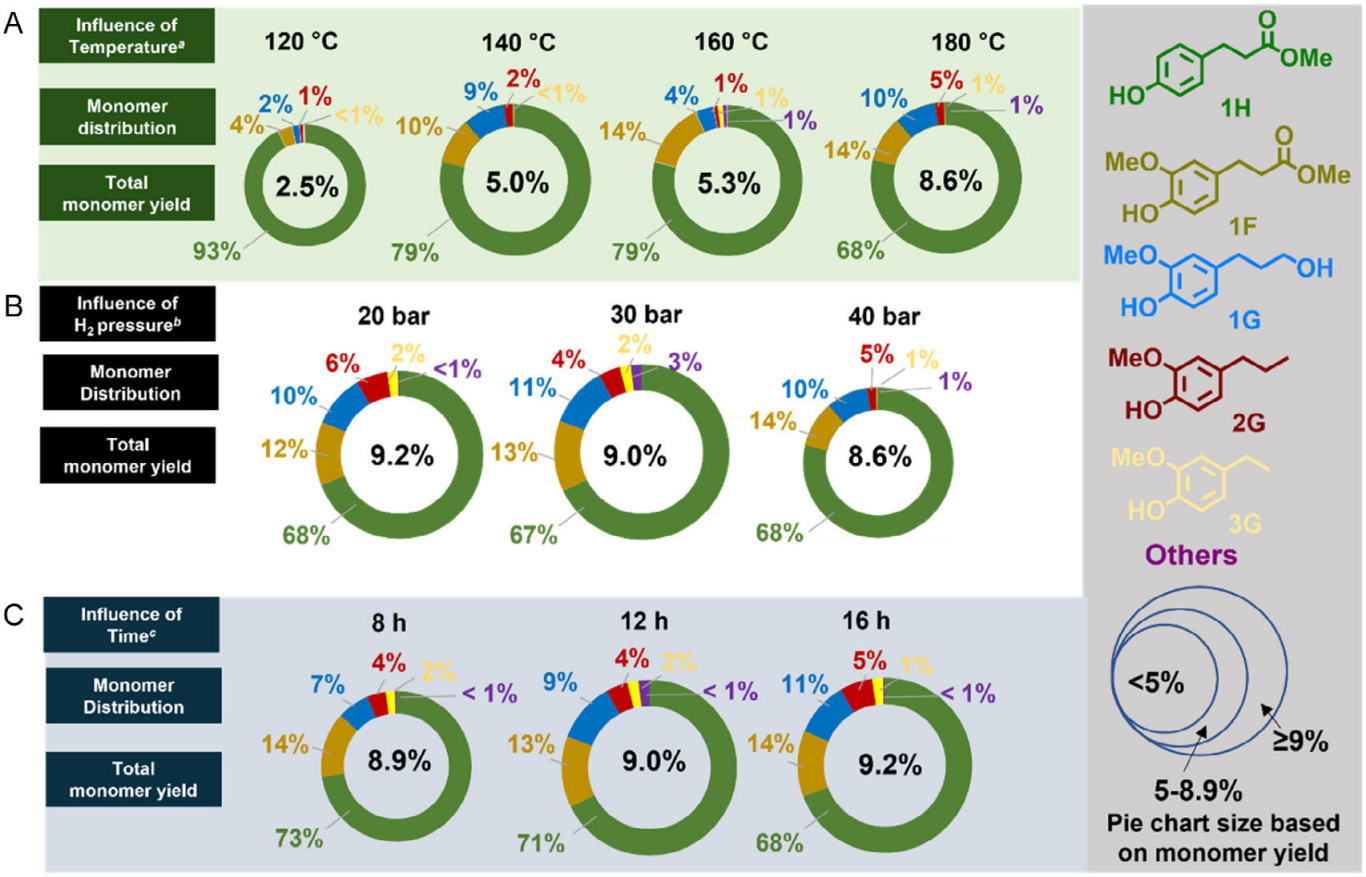
Reductive catalytic fractionation of sugarcane bagasse (1000 mg) in MeOH (20 mL) mediated by Cu_20_PMO (200 mg). General reaction conditions: (A) 40 bar H_2_, 16 h, 120–180°C. (B) 20–40 bar H_2_, 16 h, 180°C. (C) 20 bar, 8–16 h, 180°C. Monomer distribution and monomer yield determined by GC‐FID using 3,5‐dimethylphenol as internal standard. Monomer yield = weight_monomers_/weight_lignin_. For numerical values, see Tables S1–S3.

As summarized in Figure 3A, preliminary fractionation studies were performed starting from 1 g of sugarcane bagasse, Cu_20_PMO (25 wt%) and MeOH as both solvent and esterification agent, in a reductive atmosphere (H_2_ = 40 bar) at *T* = 120–180°C for *t* = 16 h.

A monomer yield of 8.6 wt% was achieved when the reaction was performed at 180°C, obtaining **1H** as the major compound with good selectivity (68%). Lower monomer yield was detected when the RCF treatment was carried out at 160°C, observing a similar outcome when the process was evaluated at 140°C (5.0–5.3 wt% yield), although a significantly higher selectivity toward **1H** (79%) was observed in both cases. As expected, a substantial decrease in the monomer yield (2.5 wt%) was observed at 120°C, despite the remarkably high selectivity toward **1H** under such conditions (93%). This provides valuable insight on the stronger association of **1H** with lignin, whereas ferulic acid is mainly esterified with hemicellulose methyl (**1F**), but also on the ability of Cu_20_PMO to selectively deconstruct lignocellulose. Nevertheless, it is conceivable that the presence of basic sites in the Cu_20_PMO could also induce further decarboxylation and hydrogenation reactions leading to the production of *para*‐alkane substituted phenols such as **3G/H** (Figure 2), phenomena observed during the RCF of herbaceous feedstocks at high temperatures (>200°C) and hydrogen pressure >30 bar [19, 20]. Therefore, the influence of hydrogen pressure on the monomer distribution is presented in Figure 3B, observing an analogous outcome when the RCF treatment is performed employing a hydrogen pressure ranging from 20 to 40 bar.

Next, the performance of the methodology was evaluated in terms of reaction time, observing also comparable monomer yields ranging between 8.9 and 9.2% when the RCF treatment was performed either during 8, 12, or 16 h (Figure 3C), delivering **1H** as major compound in all the cases (73% when the reaction was carried out for 8 h), detecting methyl ferulate (**1F**) and 4‐propanol guaiacol (**1G**) as side products along with traces of 4‐propyl guaiacol (**2G**) and other phenolics (as displayed in Figure S1). Satisfactorily, these yields are comparable to those previously reported employing Ru‐based protocols [34]. As previously demonstrated in the case of softwood, Cu_20_PMO enables the production of lignin monomer fractions containing predominantly one isolable aromatic compound. To our delight, the isolation of **1H** was successfully conducted via flash chromatography affording an isolated 5.2 wt% yield on lignin basis (15.4 mg), nevertheless, it is acknowledged that further tuning of the depolymerization step could lead to the production of lignin monomers in higher yield, although at a possible expense of decreasing the selectivity toward **1H**, therefore, a more judicious selection of optimal reaction conditions, especially considering the use of acid cocatalysts (e.g. H_3_PO_4_) which have been previously demonstrated a cooperative effect on the RCF of herbaceous feedstocks [17, 18].

### 2.2 Catalytic Amination of Glycerol Carbonate (GlyC) with Isopropyl Amine: Synthesis of GlyCa

Cyclic organic carbonates [35] are prominent renewable building blocks with wide‐ranging applications as bio‐derived nontoxic solvents, energy carriers for Li‐ion batteries as well as intermediates for the production of diverse chemicals including polymers and surfactants [36, 37, 38, 39, 40]. In particular, **GlyC** is the most well‐known renewable base cyclic organic carbonate, which can be synthesized from CO_2_ and bio‐diesel derived glycerol [41]. Moreover, the reaction of cyclic organic carbonates with phenols could enable the sustainable production of important active pharmaceutical ingredients (API's), namely, guaifenesin, methocarbamol, DPP, or 2‐hydroxymethyl‐1‐4‐benzodioxane [41, 42]. Indeed, the emerging application of cyclic organic carbonates for the alkylation of phenols allows atom‐efficient transformations, avoiding the usage of alkyl halides [43], therefore, we envisioned **GlyC** as an alternative sustainable methodology for the alkylation of lignin derived **1H** with **GLyC**, a sustainable analogous of toxic, petroleum derived epichlorohydrin.

**FIGURE 4:**
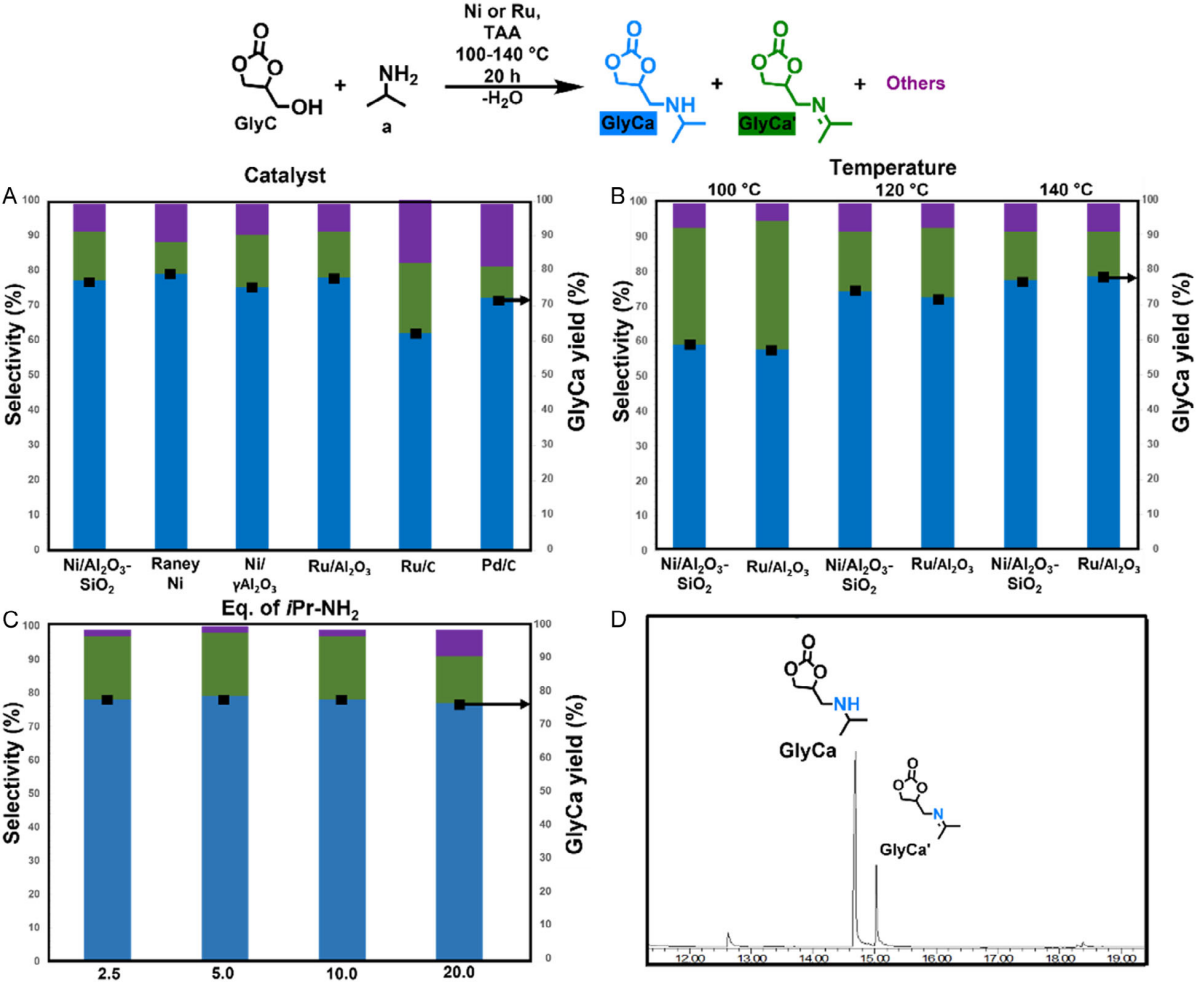
Catalytic amination of glycerol (**GlyC**) carbonate with isopropyl amines. General reaction conditions: glycerol carbonate (**GlyC**, 0.5 mmol), isopropylamine (**a**, 1.25–10 mmol), catalyst (50 mg), *tert*‐amyl alcohol (TAA, 2.5 mL), 100–140°C, 20 h. (A) Evaluation of catalyst on the selectivity and **GlyCa** yield. (B) Influence of temperature. (C) Evaluation of equivalents of isopropylamine on the selectivity and **GlyCa yield**. (D) GC‐FID traces of catalytic amination containing **GlyCa** as main product. Selectivity and conversion were determined by GC‐FID based on the peak area using dodecane as internal standard. For numerical values see Tables S4–S6.

As previously described, the *β*‐amino alcohol motif can be incorporated on **GlyC** upon catalytic amination with isopropyl amine, occurring via hydrogen bonding [44], followed by the subsequent phenol oxoalkylation of **1H** with the aminated derivative **GlyCa**, forming CO_2_ as the only coproduct. Indeed, the animation of glycerol‐derived products, such as *solketal*, through borrowing hydrogen protocols, has been earlier reported by Kann and coworkers, using Ru‐based homogenous systems [45]. Interestingly, our group has also previously demonstrated the amination of diverse biomass‐derivable alcohols mediated by earth‐abundant Raney Ni and Ni/Al_2_O_3_‐SiO_2_ catalysts using *tert*‐amyl alcohol (TAA) as bio‐based solvent and diverse ammonia sources at temperatures ranging 160–180°C [32, 46]. In addition, conventional use of Ru and Pd‐based heterogeneous catalysts has also facilitated the amination of a myriad of biomass‐derived oxygenates [47, 48]. Therefore, based on these antecedents, we conducted the evaluation of a set of Ni, Ru and Pd‐based heterogenous catalysts for the amination of **GlyC** (0.5 mmol) in TAA employing 0.4 mL of isopropyl amine (**1a**) at 140°C during 20 h, as depicted in Figure 4A.

Gratifyingly, full conversion of **GlyC** was afforded in all cases, detecting the formation of **GlyCa** as the main product along with imine derivative **GlyCa’** and other side products. A higher selectivity toward **GlyCa**/**GlyCa’** was nonetheless observed when the reaction was mediated either by Ni/Al_2_O_3_–SiO_2_, Raney Ni, or Ru/Al_2_O_3_. Therefore, we decided to continue our study employing the above‐mentioned catalysts, except for Raney Ni, due to its disadvantageous instability in air.

The hydrogenation of imine **GlyCa’** toward the desired product **GlyCa** proceeds more efficiently at high temperatures, as demonstrated when the reaction was performed at 140°C (Figure 4B), observing an increase of imine intermediate **GlyCa’** at 120°C and 100°C, respectively.

Although the efficient amination of alcohols with aliphatic amines has been previously reported employing higher temperatures (*T* = 160–200°C) [48, 49, ], cyclic carbonates such as **GlyC** typically undergo extensive decarboxylation under those conditions [39], potentially leading to undesired side products; therefore, further optimization was carried out at *T* ≤ 140°C.

Finally, different amounts of isopropyl amine were evaluated employing both Ni/Al_2_O_3_–SiO_2_ and Ru/Al_2_O_3_ catalysts at 140°C during 20 h (See Supplementary Information, Table S6). Full conversion and high selectivity (78%) toward **GlyC** were achieved with the usage of Ni/Al_2_O_3–_SiO_2_ (84.7 wt%), with isopropyl amine loading decreased from 20 to 2.5 eq. (Figure 4C), as confirmed by gas chromatography GC‐FID (Figure 4D) analysis, affording **GlyCa** in a 67% isolated yield after flash chromatography.

### 2.3 Oxoalkylation of Methyl Coumarate (1H) with GlyCa: Synthesis of Esmolol

The oxoalkylation of phenols with cyclic carbonates, typically base‐mediated, allows more sustainable etherification processes [50] and, in this case, the incorporation of the *β*‐amino alcohol motif toward the synthesis of esmolol (**1Ha**). The use of strong bases such as NaOMe [40] or 1,8‐diazabicyclo(5.4.0)undec‐7‐ene (DBU) [51] are commonly used as catalysts for this reaction, however, the use of much cheaper, harmless bases such as K_2_CO_3_ or NaOH have also demonstrated good efficiency. Thus, the oxoalkylation of **1H** was carried out employing 2 eq. of **GlyCa** with K_2_CO_3_ (5 mol%) and DMF (0.05 mL) as additives at 160°C (See Table 1). Gratifyingly, full conversion was acquired after 16 h of reaction, observing high selectivity toward the formation of **1Ha**, affording a 79% yield according to GC‐FID analysis (Table 1, entry 1).

**TABLE 1:** Oxoalkylation of **1H** with **GlyCa**: Synthesis of esmolol (**1Ha**)^a^.

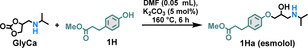		
Entry	Deviation of standard conditions	**1Ha** (%)^b^
**1**	—	79 (58)^c^
**2**	Without K_2_CO_3_	24
**3**	NaOH instead of K_2_CO_3_	65
**4**	TBAF instead of K_2_CO_3_	76
**5**	*T* = 150°C	54
**6**	Without DMF	36
**7**	0.2 mL of DMF	19

a General reaction conditions: **GlyCa** (0.5 mmol), **1H**, (0.25 mmol), DMF (0.05 mL), additive (0.025 mmol), 170°C, 16 h.

b Determined by GC‐FID using dodecane as internal standard.

c Isolated yield.

The mandatory use of a base catalyst was demonstrated when the reaction was carried out in the absence of K_2_CO_3_, observing a drastically lower yield of **1Ha** (24%) (Table 1, entry 2). On the other hand, a similar outcome as in the case of K_2_CO_3_ was detected when NaOH or TBAF were utilized as additives (Table 1, entries 3–4). Moreover, lower yields were achieved when the reaction temperature was decreased to 150°C, insufficient for the activation of the cyclic carbonate, affording only a 54% yield when the reaction was performed at 150°C (entry 5). Finally, a detrimental influence was also denoted when the reaction is carried out either under neat conditions (Table 1, entry 6) or when a higher amount of DMF was employed, observing in this case the presence of decarboxylation side products (Table 1, entries 7). Nonetheless, future studies should consider the replacement of toxic DMF by using more environmentally friendly solvents including *γ*‐valerolactone, 2‐methyltetrahydrofuran, or CPME, especially when targeting molecules with pharmaceutical relevance. The oxoalkylation of **1H** is initially promoted the base via deprotonation, inducing a nucleophilic attack on the activated soft electrophilic sites of the **GlyC** ring, namely the less hindered site, where the ring opening of the cyclic carbonate takes place. Finally, further decarboxylation of intermediate **1H’** leads to the formation of esmolol (**1Ha**) (Figure S2).

### 2.4 Evaluation of Green Credentials for the Synthesis of Esmolol (1 Ha)

The *greenness* of the developed methodology was qualitatively evaluated in terms of the CHEM21 Green Metrics Toolkit [52, 53] at the first pass level (Table 2). In the case of solvent type, the use of MeOH during the RCF step results highly advantageous toward the formation of **1H;** however, it can also be problematic from a health and safety perspective. Instead, the usage of potentially bio‐derived TAA during the amination step significantly improves the *greenness* of the reaction. Regarding the oxoalkylation step, although the use of DMF was required in order to increase the yield (Table 1, entry 6), less hazardous and more environmentally friendly alternatives would be nonetheless highly desired.

**TABLE 2:** Qualitative assessment of solvent use, inherent hazards of used chemicals, catalyst, or reagent use, energy and workup methods for the synthesis of **1Ha** from sugarcane bagasse.

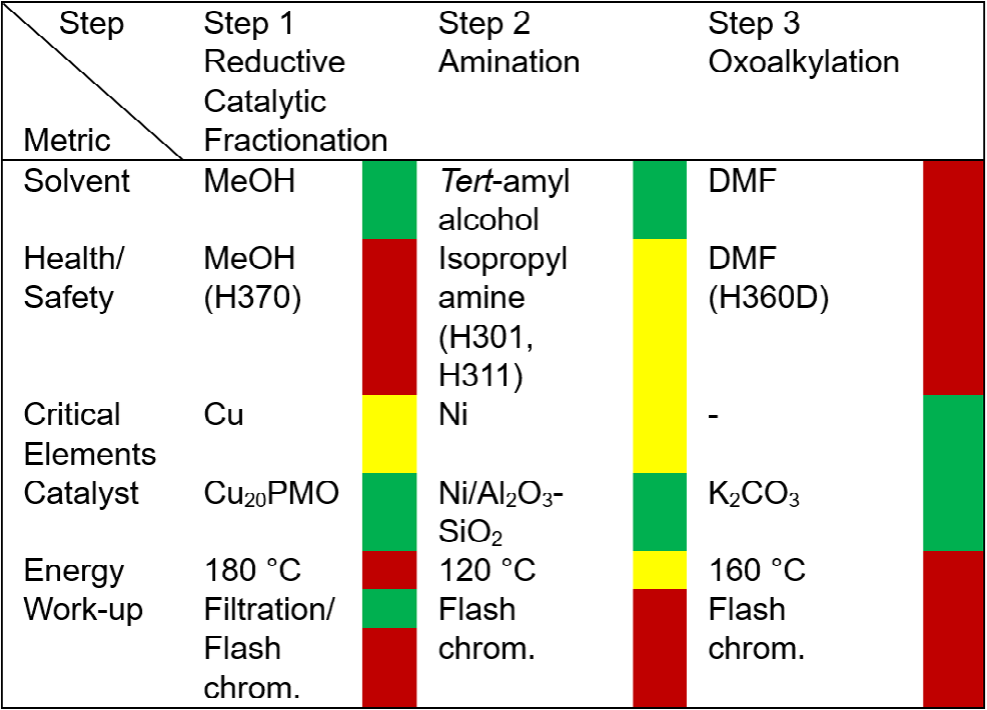

Satisfactorily, the use of earth‐abundant catalysts (Cu_20_PMO and Ni/Al_2_O_3_–SiO_2_) were efficiently employed during the RCF and amination step, avoiding the use of highly critical elements. Generally, energy intensive conditions were required for lignocellulose fractionation (Step 1) as well as for the alkylation of phenols with organic carbonates, whereas the amination of **GlyC** with isopropyl amine (Step 2) was afforded under considerably milder reaction conditions compared to glycerol and other glycerol‐derived building blocks (e.g., propane‐1,2‐diol).

## 3 Conclusion

Deriving value from agricultural waste by catalytic strategies targeting the production of pharmaceutically‐relevant products provide an attractive alternative to maximize the potential of bio‐based platform aromatic chemicals. Here, a straightforward and atom‐economic methodology toward the synthesis of *β*‐adrenergic blocker esmolol (**1Ha**) from sugarcane bagasse has been developed. First, the depolymerization of sugarcane via a lignin‐first RCF process catalyzed by Cu_20_PMO under mild conditions allowed for the selective sourcing of methyl(3‐(3‐hydroxy‐4‐methoxyphenyl)propionate (**1H**) in a 5.2 wt% yield on lignin basis. On the other hand, the catalytic amination of **GlyC**, a prominent coproduct of the biodiesel industry, has been studied through the hydrogen borrowing methodology using earth‐abundant catalysts such as Ni/Al_2_O_3_–SiO_2_, affording the construction of intermediate of **GlyCa**. The combination of these two bio‐based platform chemicals, methyl(3‐(3‐hydroxy‐4‐methoxyphenyl)propionate (**1H**) and amine‐functionalized glycerol carbonate (**GlyCa**), allows a clean synthetic pathway toward **1Ha** with only H_2_O and CO_2_ as the main side products. Merging both biomass valorization and the use of catalytic approaches, this method contributes to the development of clean synthetic strategies for the sustainable synthesis of pharmaceuticals.

## 4 Experimental Section

### 4.1 General Information

Sugarcane bagasse samples were provided by Dr. Henrique Brasil (State University of Campinas, Brazil) and employed without prior treatment. All chemicals and reagents were obtained from commercial sources and used without further purification. Flash chromatography was carried out using silica gel (200–300 mesh) and typically cyclohexane and ethyl acetate as eluent.

### 4.2 Characterization

GC was used for products identification as well as the determination of conversion and selectivity values. Products identification was performed by gas chromatography‐mass spectometry (GC–MS 5975C MSD) equipped with an HP‐5 MS column, and helium as carrier gas. The temperature program started at 50°C for 5 min, heated by 10°C min^−1^ to 325°C and held for 5 min. Conversion and products selectivity were determined by gas chromatography‐flame ionization detector (GC‐FID Shimadzu Agilent 8890 GC) equipped with an HP‐5 MS column using nitrogen as carrier gas. Nuclear magnetic resonance (NMR) spectroscopy: ^1^H, and ^13^C NMR spectra were recorded on a Bruker Avance III 300 MHz (300 and 75 MHz, respectively. ^1^H and ^13^C NMR were recorded at RT. Chemical shift values are reported in ppm with the solvent resonance as the internal standard (CDCl_3_: 7.26 for ^1^H, 77.0 for ^13^C; CD_3_OD: 3.31 for ^1^H, 49.0 for ^13^C; DMSO‐d6: 2.50 for ^1^H, 39.5 for ^13^C). Data are reported as follows: chemical shifts, multiplicity (*s* = singlet, *d* = doublet, *t* = triplet, *q = *quartet, br. = broad, *m* = multiplet), coupling constants (Hz), and integration.

### 4.3 Catalyst Preparation

Cu_20_PMO catalyst was prepared according to our previously reported procedure [28]. In a typical procedure, a solution containing AlCl_3_ · 6H_2_O (12.07 g, 0.05 mol), Cu(NO_3_)_2_·2.5H_2_O (6.98 g, 0.03 mol) and MgCl_2_ · 6H_2_O (24.4 g, 0.12 mol) in deionized water (200 mL) was dropwise added to a solution containing Na_2_CO_3_ (5.30 g, 0.05 mol) in water (300 mL) at 60°C under vigorous stirring. The pH value was always kept between 9 and 10 by the addition of small portions of a 1 M solution of NaOH. The mixture was vigorously stirred at 60°C for 72 h. After cooling to room temperature, the light blue solid was filtered and resuspended in a 2 M solution of Na_2_CO_3_ (300 mL) and stirred overnight at 40°C. The catalyst precursor was filtered and washed with deionized water until chloride free. After drying the solid for 6 h at 100°C followed by the calcination at 460°C for 24 h in air, 9.5 g of Cu_20_PMO was obtained.

## 5 Methods

### 5.1 Reductive Catalytic Fractionation of Sugarcane Lignocellulose in MeOH

The mild depolymerization of sugarcane bagasse was carried out in a high‐pressure Parr autoclave equipped with an overhead stirrer. Typically, the autoclave was charged with 0.2 g of Cu_20_PMO catalyst, 1.0 g of sugarcane lignocellulose and MeOH (20 mL) as solvent. Then, the reactor was sealed and pressurized with H_2_ at room temperature. Subsequently, the reactor was heated at the given temperature and stirred at 400 rpm for 18 h. After completion of the reaction, the reactor was cooled to room temperature. Then 0.1 mL solution was collected through a syringe and injected to GC–MS or GC‐FID after filtration through a PTFE filter (0.45 μm). The solid was separated from the solution by centrifugation and subsequent decantation and additionally washed with MeOH (3 × 20 mL). Finally, MeOH extracts were combined in a round bottom flask and the solvent was removed in vacuo.

### 5.2 Catalytic Amination of Glycerol Carbonate (**Glyc**) with Isopropylamine

Typically, a 20 mL microwave glass vial was charged with 0.5 mmol **GlyC**, 1.25–10 mmol isopropylamine (**a**), 50 mg of catalyst, and 2.5 mL of t‐amyl alcohol as solvent. Then, the vial was sealed with a silver aluminum/silicon crimp cap and placed into a heating block and heated at the indicated temperature, typically 100–140°C and stirred at 400 rpm. After completion of the reaction, the vial was cooled down to RT, the solution was filtered and 0.1 mL solution was collected through a syringe and injected to GC–MS or GC‐FID through a PTFE filter (0.45 μm) and the isolation of the desired product was carried out by flash chromatography.

### 5.3 Oxoalkylation of 3‐(4‐hydroxyphenyl) Propionate (**1H**) with **GlyCa**

Typically, a 20 mL microwave glass vial was charged with 0.5 mmol of **1H** (0.25 mmol), **GlyCa** (0.5 mmol) and an additive (0.025 mmol). Then, the vial was sealed with a silver aluminum/silicone crimp cap and placed into a heating block and heated at the indicated temperature, typically 160°C and stirred at 400 rpm. After completion of the reaction, the vial was cooled down to RT, the crude mixture was solubilized in EtOAc (5 mL) and filtered through a PTFE filter and a 0.1 mL aliquot was taken for GC–MS and GC‐FID analysis. Finally, the isolation of the desired product was carried out via flash chromatography.

## Supporting Information

Additional supporting information can be found online in the Supporting Information section. The authors have cited additional references within the Supporting Information [25, 26, 49, 50]. Additional supporting information can be found online in the Supporting Information Section. **Supporting Fig. S1:** GC‐FID traces of Supplementary Table 3, Entry 1. Reaction conditions: Sugarcane Bagasse (1000 mg), Cu_20_PMO (200 mg), MeOH (20 mL), 20‐40 bar H_2_, 16 h, 180 °C. **Supporting Fig. S2:** Proposed reaction mechanism for the production of Esmolol (**1Ha**) with **GlyCa**. **Supporting Fig. S3:** ^1^H and ^13^C NMR spectra (CDCl_3_) of **GlyCa**. **Supporting Table S1:** Mild reductive catalytic fractionation of sugarcane lignocellulose: Influence of temperature^[a]^. **Supporting Table S2:** Mild reductive catalytic fractionation of sugarcane lignocellulose: Influence of hydrogen pressure^[a]^. **Supporting Table S3:** Mild reductive catalytic fractionation of sugarcane lignocellulose: Influence of time of reaction^[a]^. **Supporting Table S4:** Catalytic amination of Glycerol Carbonate (**GlyC**) with isopropyl amine (**a**): Evaluation of catalys^[a]^. **Supporting Table S5:** Catalytic amination of Glycerol Carbonate (**GlyC**) with isopropyl amine (**a**): Evaluation of temperature^[a]^. **Supporting Table S6:** Catalytic amination of Glycerol Carbonate (**GlyC**) with isopropyl amine (**a**): Evaluation of temperatur^[a]^.

## Funding

This research was funded in whole, or in part, by the Austrian Science Fund (FWF) https://doi.org/10.55776/PAT5229224. For the purpose of open access, the author has applied a CC BY public copyright licence to any Author Accepted Manuscript version arising from this submission

## Conflicts of Interest

The authors declare no conflicts of interest.

## Supporting information

Supplementary Material

## Data Availability

The data that support the findings of this study are available from the corresponding author upon reasonable request.
