# Supplementary material for: Merging Lignin and Glycerol Carbonate Valorization Toward the Green Synthesis of β‐Adrenergic Blocker Esmolol
Source: ChemSusChem. 2025 Dec 14;19(1):e202501540. doi: 10.1002/cssc.202501540 (PMC12767559; doi:10.1002/cssc.202501540)
Supplement: Supplementary file 1 — Supplementary Material [file CSSC-19-e202501540-s001.pdf]

1. General methods

Sugarcane bagasse samples were provided by Dr. Henrique Brasil (State University of Campinas, Brazil) and employed without prior treatment. All chemicals and reagents were obtained from commercial sources and used without fur-ther purification. Flash chromatography was carried out using silica gel (200-300 mesh) and typically cyclohexane and ethyl acetate as eluent.

Column chromatography was performed using Merck silica gel type 9385 230-400 mesh and typically pentane and ethyl acetate as eluent.

Thin layer chromatography (TLC): Merck silica gel 60, 0.25 mm. The components were visualized by UV or $KMnO_4$ staining.

Analytical methods. Product identification was performed by GC-MS (Shimadzu QP2010 Ultra) with an HP-1MS column, and helium as carrier gas. GC-MS method: The temperature program started at 50 °C for 5 min, heated by 30 °C/minute to 250 °C and held for 15 min. Conversions and product selectivity were determined by GC-FID (Agilent Technologies 6890) with an HP-5MS column using nitrogen as carrier gas. GC-FID analysis method: The temperature program started at 50 °C for 5 min, heated by 30 °C/min to 320 °C and held for 15 min. ^1H and ^{13}C NMR spectra were recorded on a Varian Mercury Plus 400, Agilent MR 400 (400 and 101 MHz, respectively), Varian Inova 500 (500 and 126 MHz, respectively) and Bruker Avance NEO 600 (600 and 151 MHz, respectively) using $CDCl_3$ as a solvent. ^1H and ^{13}C NMR spectra were recorded at room temperature. Chemical shift values are reported in ppm with the solvent resonance as the internal standard ($CDCl_3$: 7.26 for 1H, 77.00 for ^{13}C). Data are reported as follows: chemical shifts, multiplicity (s = singlet, d = doublet, t = triplet, q = quartet, br. = broad, m = multiplet), coupling constants (Hz), and integration.

1.1 Preparation and characterization of Cu-PMO catalyst

The HTC (hydrotalcite) catalyst precursors were prepared by a co-precipitation method, according to literature.[1] The catalyst prepared in this procedure is named as Cu20-PMO in which 20% of the Mg^{2+} ions were replaced with Cu^{2+} ions in a 3:1 Mg/Al hydrotalcite precursor. In a typical procedure, a solution containing $AlCl_3{\cdot}6H2O$ (12.07 g, 0.05 mol), $Cu(NO_3)_2{\cdot}2.5H_2O$ (6.98 g, 0.03 mol) and $MgCl_2{\cdot}6H_2O$ (24.40 g, 0.12 mol) in deionized water (0.2 L) was added to a solution containing Na_2CO_3 (5.30 g, 0.05 mol) in water (0.3 L) at 60 o C under vigorous stirring. The pH was kept between 9 and 10 by addition of small portions of a 1 M solution of NaOH. The mixture was vigorously stirred at 60 o C for 72 h. After cooling to room temperature, the light blue solid was filtered and resuspended in a 2 M solution of Na_2CO_3 (0.3 L) and stirred overnight at 40 ˚C. The catalyst precursor was filtered and washed with deionized water until chloride free. After drying the solid for 6 h at 100 o C, 15.07 g of the hydrotalcite (HTC) was obtained. Before use, 4 g of hydrotalcite was calcined at 460 ˚C for 24 h in air and 2.5 g of Cu20-PMO can be obtained.

2. General experimental procedures

2.1 Reductive Catalytic Fractionation of Sugarcane Lignocellulose[2]

General Procedure: The mild depolymerization of sugar cane bagasse was carried out in a 100 mL high pressure Parr autoclave with an overhead stirrer. The autoclave was charged with Cu20-PMO catalyst (0.2 g), sugarcane lignocellulose (1 g), 3,5-dimethylphenol (20 mg as internal standard) and methanol (20 mL). Then, the reactor was sealed and pressurized with H_2 (40 bar) at room temperature. The reactor was heated at the desired temperature (120-180 ˚C) and stirred at 400 rpm. After reaction, the reactor was cooled down to room temperature. Then 0.1 mL solution was collected with syringe and injected to GC-MS and GC-FID after filtration with a PTFE filter (0.42 μm). After that the solution and solids were transferred into a 50 mL centrifuge tube. The solid was separated from the reaction solution by centrifugation and subsequent decantation, additionally washed with methanol (2 × 40 mL), and dried overnight in the desiccator under vacuum. All the solution was collected in a round bottom flask and the solvent was removed. The remaining products were dried with rotary evaporation under vacuum. Analysis of the liquid sample was performed on a Hewlett Packard 6890 series equipped with a HP-5 capillary column and a flame ionization detector (FID). The following operating conditions were used: injection temperature of 300 ˚C, column temperature program: 40 ˚C (5 min), 10 ˚C /min to 280 ˚C (6 min), detection temperature of 300 ˚C.

Quantification of the lignin monomers was performed as follows: Sensitivity factors of the products were obtained by calibration with authentic standards. Identification of lignin monomers was first performed on GC-MS and then confirmed by comparing with authentic standards

Table S1.
Mild reductive catalytic fractionation of sugarcane lignocellulose: *Influence of temperature*[a]

**Entry**		**1**	**2**	**3**	**4**
**Sugarcane bagasse** (mg)		1000	1000	1000	1000
**T** (°C)		**120**	**140**	**160**	**180**
**H_2** (bar)		40	40	40	40
**MeOH** solubles (mg)		76	93	114	189
**Monomers** (mg)[b]		7.4	15.0	15.6	25.8
**Monomer yield** (%)[c]		2.5	5.0	5.3	8.6
**Monomer Distribution** (%)[d]	**1H**	93	79	79	68
	**1F**	4	10	14	14
	**1G**	2	9	4	10
	**2G**	1	2	1	5
	**3G**	<1	<1	1	1
	**Others**	<1	<1	1	1
**1H** (mg)[b]		**6.9**	**11.9**	**12.3**	**17.4**

[a] General reaction conditions: Sugarcane Bagasse (1000 mg), $Cu_{20}PMO$ (200 mg), 40 bar H_2, MeOH (20 mL), 16 h, 120-180 °C. [b] Determined by GC-FID (calibrated) using 3,5-dimethylphenol as internal standard. [c]Monomer yield= $weight_{monomers}/weight_{lignin}$. [d] Products: methyl 3-(4-hydroxyphenyl)propionate (**1H**), methyl 3-(3-hydroxy-4-methoxyphenyl)propionate (**1F**), 4-propanolguaiacol (**1G**), 4-propylguaiacol (**2G**), 4-ethylguaiacol (**3G**).

Table S2.
Mild reductive catalytic fractionation of sugarcane lignocellulose: *Influence of hydrogen pressure*[a]

**Entry**		**1**	**2**	**3**
**Sugarcane bagasse** (mg)		1000	1000	1000
**T** (°C)		180	180	180
**H_2** (bar)		**20**	**30**	**40**
**MeOH** solubles (mg)		133	165	189
**Monomers** (mg)[b]		27.7	26.8	25.8
**Monomer yield** (%)[c]		9.2	9.0	8.6
**Monomer Distribution** (%)[d]	**1H**	68	67	68
	**1F**	12	13	14
	**1G**	10	11	11
	**2G**	6	4	5
	**3G**	2	2	1
	**Others**	<1	3	1
**1H** (mg)[b]		**19.0**	**18.7**	**17.4**

[a] General reaction conditions: Sugarcane bagasse (1000 mg), $Cu_{20}PMO$ (200 mg), MeOH (20 mL), 20-40 bar H_2, 16 h, 180 °C. [b] Determined by GC-FID using 3,5-dimethylphenol as internal standard. [c] Monomer yield= $weight_{monomers}/weight_{lignin}$. [d] Products: methyl 3-(4-hydroxyphenyl)propionate (**1H**), methyl 3-(3-hydroxy-4-methoxyphenyl)propionate (**1F**), 4-propanolguaiacol (**1G**), 4-propylguaiacol (**2G**), 4-ethyl guaiacol (**3G**).

Table S3.

Mild reductive catalytic fractionation of sugarcane lignocellulose: *Influence of time of reaction*[a]

**Entry**		**1**	**2**	**3**
**Sugarcane bagasse** (mg)		1000	1000	1000
**T** (°C)		180	180	180
**H_2** (bar)		20	20	20
**Time** (h)		**8**	**12**	**16**
**MeOH** solubles (mg)		133	142	148
**Monomers** (mg)[b]		27.7	26.8	27.7
**Monomer yield** (%)[c]		8.9	9.0	9.2
**Monomer Distribution** (%)[d]	**1H**	73	71	68
	**1F**	14	13	14
	**1G**	7	9	11
	**2G**	4	5	5
	**3G**	2	2	1
	**Others**	<1	<1	<1
**1H** (mg)[b]		**19.9 (15.4)[e]**	**19.2**	**19.0**

[a] General reaction conditions: Sugarcane Bagasse (1000 mg), Cu_{20}PMO (200 mg), MeOH (20 mL), 20 bar H_2, *8*-16 h, 180 °C. [b] Determined by GC-FID using 3,5-dimethylphenol as internal standard. [c] Monomer yield= $weight_{monomers}/weight_{lignin}$. [d] Products: methyl 3-(4-hydroxyphenyl)propionate (**1H**), methyl 3-(3-hydroxy-4-methoxyphenyl)propionate (**1F**), 4-propanolguaiacol (**1G**), 4-propylguaiacol (**2G**), 4-ethylguaiacol (**3G**). [e]After isolation by flash chromatography.

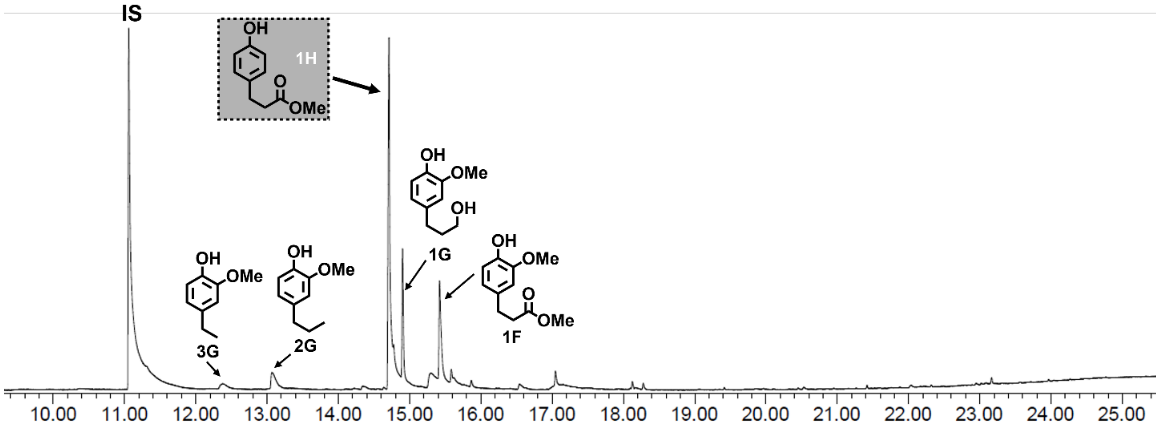

Figure S1. GC-FID traces of Supplementary Table 3, Entry 1. Reaction conditions: Sugarcane Bagasse (1000 mg), Cu_{20}PMO (200 mg), MeOH (20 mL), 20-40 bar H_2, 16 h, 180 °C.

2.2 Catalytic amination of glycerol carbonate (**GlyC**) with isopropylamine (**a**).

General Procedure: Typically, a 20 mL microwave glass vial was charged with glycerol carbonate (0.5 mmol, GlyC), isopropyl amine (a, 1.25-10 mmol), catalyst (50 mg), and *tert*-amyl alcohol (2.5 mL) as solvent. Then, the vial was sealed with a silver aluminum/silicon crimp cap and placed into a heating block and heated at the indicated tempera-ture, typically 100–140 °C and stirred at 400 rpm. After completion of the reaction, the vial was cooled down to RT, the solution was filtered and 0.1 mL solution was collected through a syringe and injected to GC-MS or GC-FID through a PTFE filter (0.45 µm) and the isolation of the desired product was carried out by flash chromatography.

Table S4.

Catalytic amination of Glycerol Carbonate (**GlyC**) with isopropyl amine (**a**): *Evaluation of catalyst*[a]

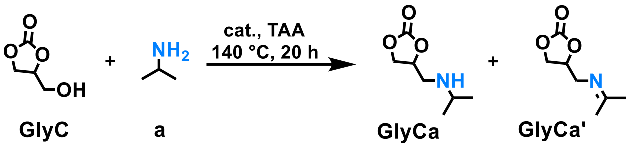

Entry	Catalyst	Conversion (%)[b]	GlyCa (%)[b]	GlyCa' (%)[b]	Others (%)[b]
**1**	Ni/Al_2O_3-SiO_2	99	77	14	8
**2**	Raney Ni	99	79	9	11
**3**	Ni/γAl_2O_3	99	75	15	9
**4**	Ru/Al_2O_3	99	78	13	8
**5**	Ru/C	99	62	20	18
**6**	Pd/C	99	72	9	18

[a]General reaction conditions: **GlyC** (59 mg, 0.5 mmol), **1a** (400 µL, 9.3 mmol), catalyst (50 mg), *tert*-amyl alcohol (**TAA, 2**.5 mL), 140 °C, 20 h. [b] Determined by GC-FID using dodecane as internal standard.

Table S5.

Catalytic amination of Glycerol Carbonate (**GlyC**) with isopropyl amine (**a**): *Evaluation of temperature* [a]

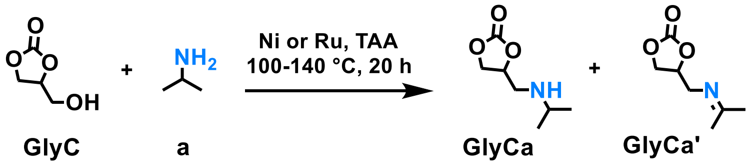

Entry	Catalyst	T (°C)	t (h)	Conversion (%)[b]	GlyCa (%)[b]	GlyCa' (%)[b]	Others (%)[b]
**1**	Ni/Al_2O_3-SiO_2	140	20	99	77	14	8
**2**	Ru/Al_2O_3	140	20	99	78	13	8
**3**	Ni/Al_2O_3/SiO_2	120	20	99	73	17	8
**4**	Ru/Al_2O_3	120	20	99	72	20	7
**5**	Ni/Al_2O_3/SiO_2	100	20	99	59	34	7
**6**	Ru/Al_2O_3	100	20	99	57	37	5

[a]General reaction conditions: **GlyC** (59 mg, 0.5 mmol), **1a** (400 µL, 9.3 mmol), catalyst (50 mg), *tert*-amyl alcohol (TAA, **2**.5 mL), 100-140 °C, 20 h. [b] Determined by GC-FID using dodecane as internal standard.

Table S6.
Catalytic amination of Glycerol Carbonate (**GlyC**) with isopropyl amine (**a**): *Evaluation of temperature* [a]

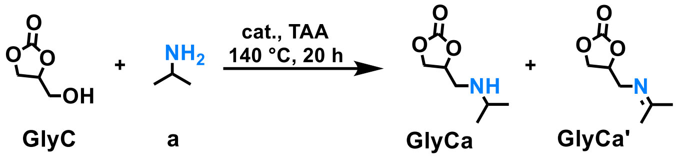

Entry	Catalyst	1a (mmol/eq)	Conversion (%)[b]	GlyCa (%)[b]	GlyCa' (%)[b]	Others (%)[b]
1	Ni/Al_2O_3-SiO_2	9.3/20	99	77	14	8
2	Ru/Al_2O_3	9.3/20	99	78	13	8
3	$Ni/Al_2O_3/SiO_2$	4.7/10	99	78	19	2
4	Ru/Al_2O_3	4.7/10	99	73	23	2
5	$Ni/Al_2O_3/SiO_2$	2.3/5	99	79	19	2
6	Ru/Al_2O_3	2.3/5	99	68	26	4
7	$Ni/Al_2O_3/SiO_2$	1.2/2.5	99	78 (67%)[c]	19	2
8	Ru/Al_2O_3	1.2/2.5	99	62	33	2

[a]General reaction conditions: **GlyC** (59 mg, 0.5 mmol), **1a** (50-400 µL, 1.2-9.3 mmol), catalyst (50 mg), *tert*-amyl alcohol (TAA, 1.5 mL),140 °C, 20 h. [b] Determined by GC-FID using dodecane as internal standard. [c]Isolated yield.

2.3 Oxoalkylation of 3-(4-hydroxyphenyl) propionate (**1H**) with **GlyCa**.
General Procedure: An oven-dried 20 mL microwave glass vial was charged with 0.5 mmol of 1H (0.25 mmol), GlyCa (0.5 mmol) and additive (0.025 mmol). Then, the vial was sealed with a silver aluminium/silicone crimp cap and placed into a heating block and heated at the indicated temperature, typically 160 °C and stirred at 400 rpm. After completion of the reaction, the vial was cooled down to RT, the crude of mixture was solubilized in EtOAc (5 mL) and filtered through a PTFE filter and a 0.1 mL aliquot was taken for GC-MS and GC-FID analysis. Finally, the isolation of the desired product was carried out via flash chromatography.

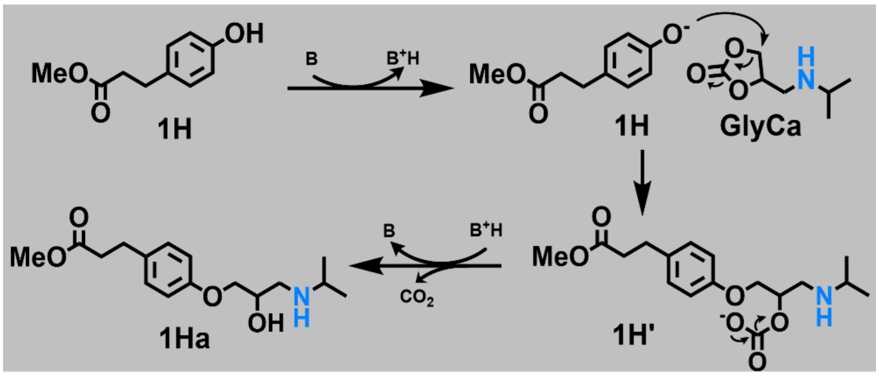

Figure S2. Proposed reaction mechanism for the production of Esmolol (**1Ha**) with **GlyCa**

3. Characterization data

4-((isopropylamino)methyl)-1,3-dioxolan-2-one (**GlyCa**)

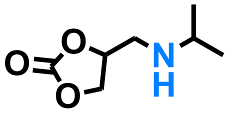

Isolated yield: 61%. **^{1}H NMR** (300 MHz, $CDCl_3$) δ 4.17 – 4.04 (m, 1H), 3.87 (q, J = 5.0 Hz, 1H), 3.82 – 3.72 (m, 2H), 3.76 – 3.50 (m, 2H), 1.14 (d, J = 6.5 Hz, 6H). **^{13}C NMR** (75 MHz, $CDCl_3$) δ 156.3, 70.7, 63.3, 62.3, 43.3, 22.9. **HRMS** (ESI+ m/z). Calculated for [M+H]+:159.178212, found:160.202341.

Methyl 3-(4-hydroxyphenyl)propanoate (**1H**)

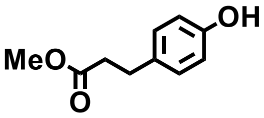

Isolated yield: 15.1 mg, 5.1 wt% on lignin basis. Spectral data are in accordance with literature.[3] **^{1}H NMR** (300 MHz, MeOD) δ 7.08 – 6.97 (m, 2H), 6.76 – 6.65 (m, 2H), 3.65 (s, 3H), 2.83 (t, J = 7.6 Hz, 2H), 2.64 – 2.53 (m, 2H). **^{13}C NMR** (75 MHz, MeOD) δ 174.1, 156.1, 132.2, 129.9, 115.9, 52.2, 36.6, 30.8. **HRMS** (ESI+ m/z). Calculated for [M+H]+: 180.364418 , found:180.809322.

Methyl 3-(4-(2-hydroxy-3-(isopropylamino)propoxy)phenyl)propanoate (**1Ha**)

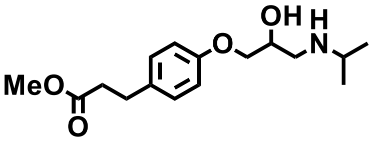

Isolated yield: 58%. Spectral data are in accordance with the literature.[4] **^{1}H NMR** (300 MHz, MeOD) δ 6.99 (d, J = 8.5 Hz, 2H), 6.72 – 6.61 (m, 2H), 4.14 – 3.94 (m, 2H), 3.72 – 3.57 (m, 2H), 3.62 – 3.49 (m, 3H), 3.55 – 3.44 (m, 2H), 2.79 (dd, J = 8.6, 6.3 Hz, 2H), 2.65 – 2.49 (m, 2H), 1.12 (dd, J = 6.6, 1.9 Hz, 6H). **^{13}C NMR** (75 MHz, MeOD) δ 174.2, 157.4, 156.9, 132.6, 130.3, 116.19, 73.8, 71.2, 66.5, 64.4, 58.2, 37.1, 31.2, 18.5. **HRMS** (ESI+ m/z). Calculated for [M+H]+:296.182944, found: 296.019847.

4. NMR Characterization of novel compound **GlyCa**

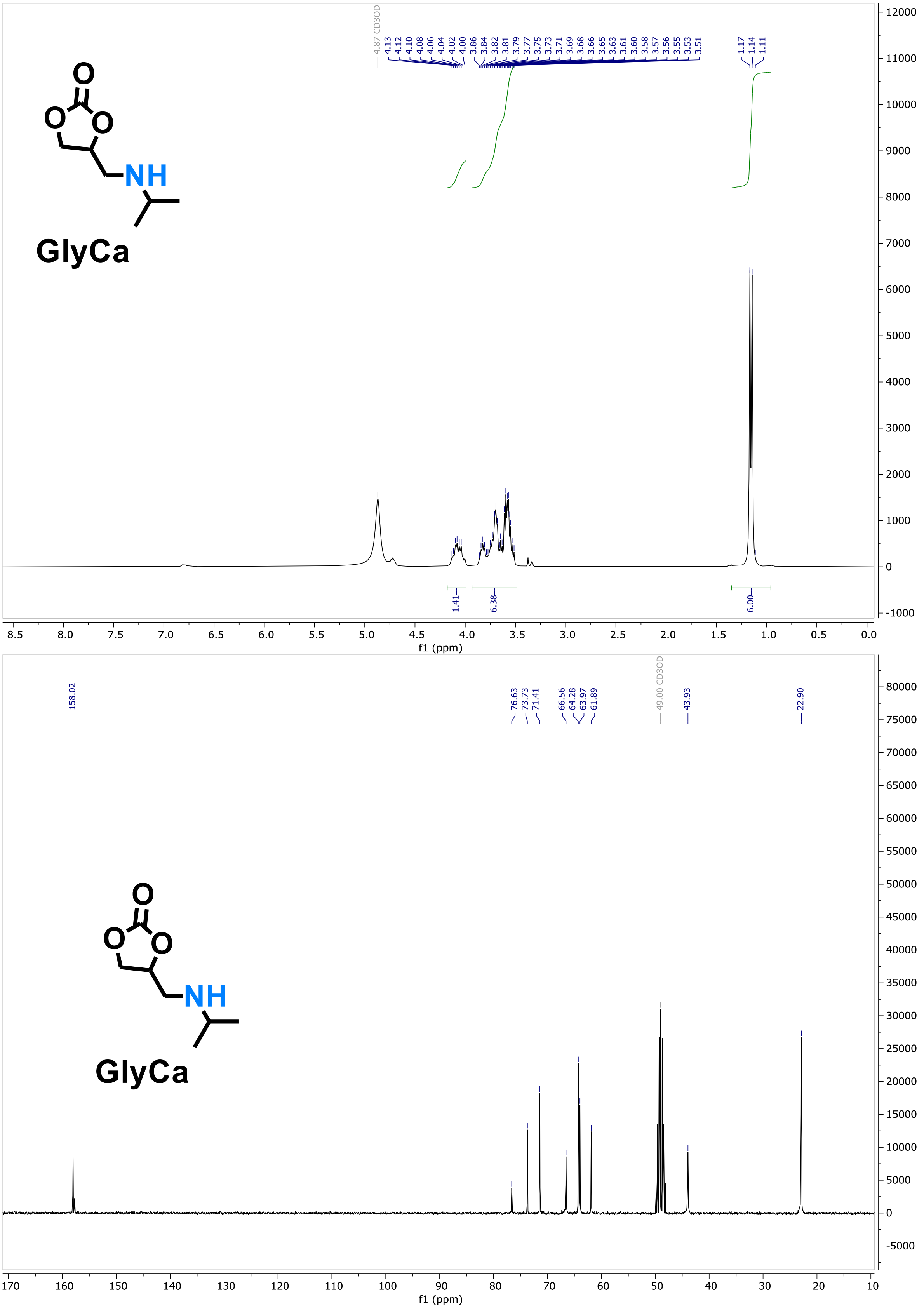

Figure S3. ^{1}H and ^{13}C NMR spectra ($CDCl_3$) of **GlyCa**

5. References

[1] K. Barta, G. R. Warner, E. S. Beach and P. T. Anastas, *Green Chem.*, **2013**, 16, 191–196.
[2] Z. Sun, G. Bottari, A. Afanasenko, M. C. A. Stuart, P. J. Deuss, B. Fridrich and K. Barta, *Nat. Catal.*, **2018**, 1, 82-92.
[3] T. Bozzini, G. Botta, M. Delfino, S. Onofri, R. Saladino, D. Amatore, R. Sgarbanti, L. Nencioni and A. T. Palamara, *Bioorg. Med. Chem*. **2013**, 21, 7699-7708
[4] Y. Wei, H. Tang, X. Cong, B. Rao, C. Wu and X. Zeng, *Org. Lett.* **2014**, 16, 2248-2251
